# Supplementary figures and images for: Single-nucleus transcriptomics of wing sexual dimorphism and scale cell specialization in sulphur butterflies
Source: PLoS Biol. 2025 Jun 18;23(6):e3003233. doi: 10.1371/journal.pbio.3003233 (PMC12204629; doi:10.1371/journal.pbio.3003233)

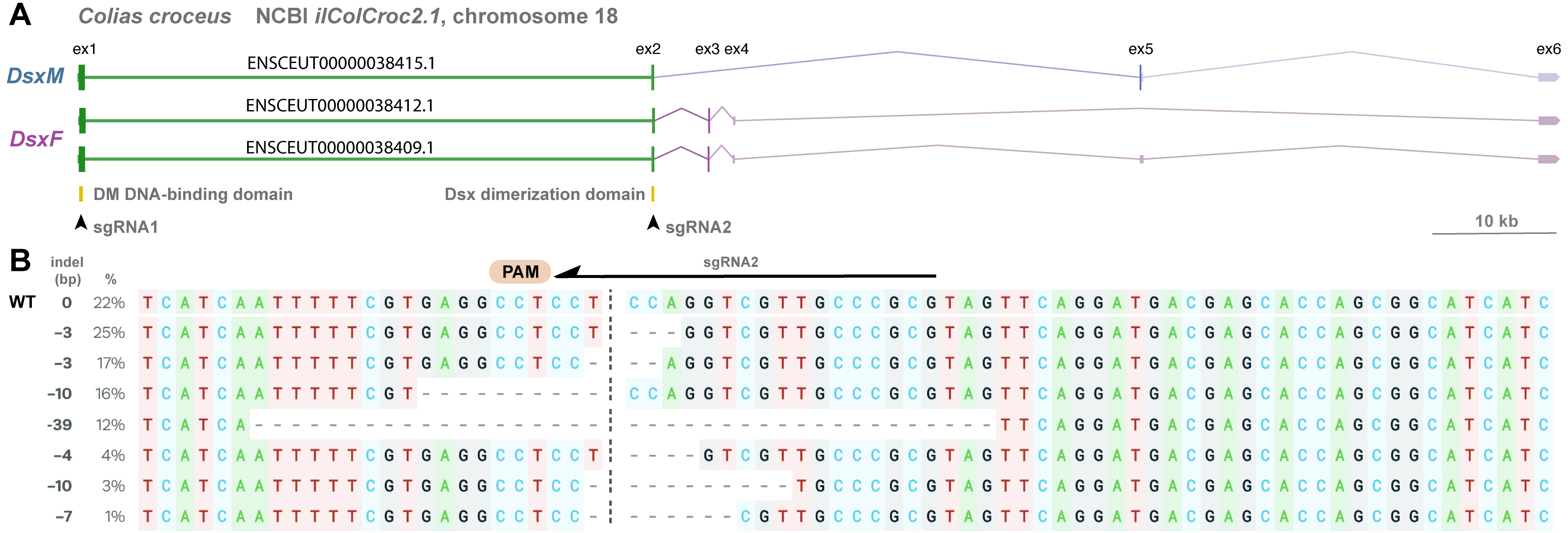

Supplement: S1 Fig — A. Overview of the Dsx locus in the Colias croceus genome annotation. The gene structure is inferred from RNAseq intron-spanning reads available on the NCBI Genome Browser, and features three major isoforms. The male isoform (DsxM, ENSCEUT00000038409.1) spans an open-reading frame on exons 1, 2 and 5. The female isoforms (DsxF, ENSCEUT00000038412.1 and ENSCEUT00000038415.1) both span an open-reading frame on exons 1, 2 and 3. CRISPR sgRNA targets were designed on the matching version of the C. eurytheme genome (arrowheads) and predicted to impact all isoforms. The exon 1 target overlaps with the region encoding the DM DNA binding domain of Dsx, while the exon 2 targets corresponds to the Dsx dimerization domain. B. Genotyping of a mosaic crispant following the targeting of Dsx exon2 using Synthego ICE chromatogram deconvolution. Dotted line: predicted cut site. (TIF) [file pbio.3003233.s001.tif]

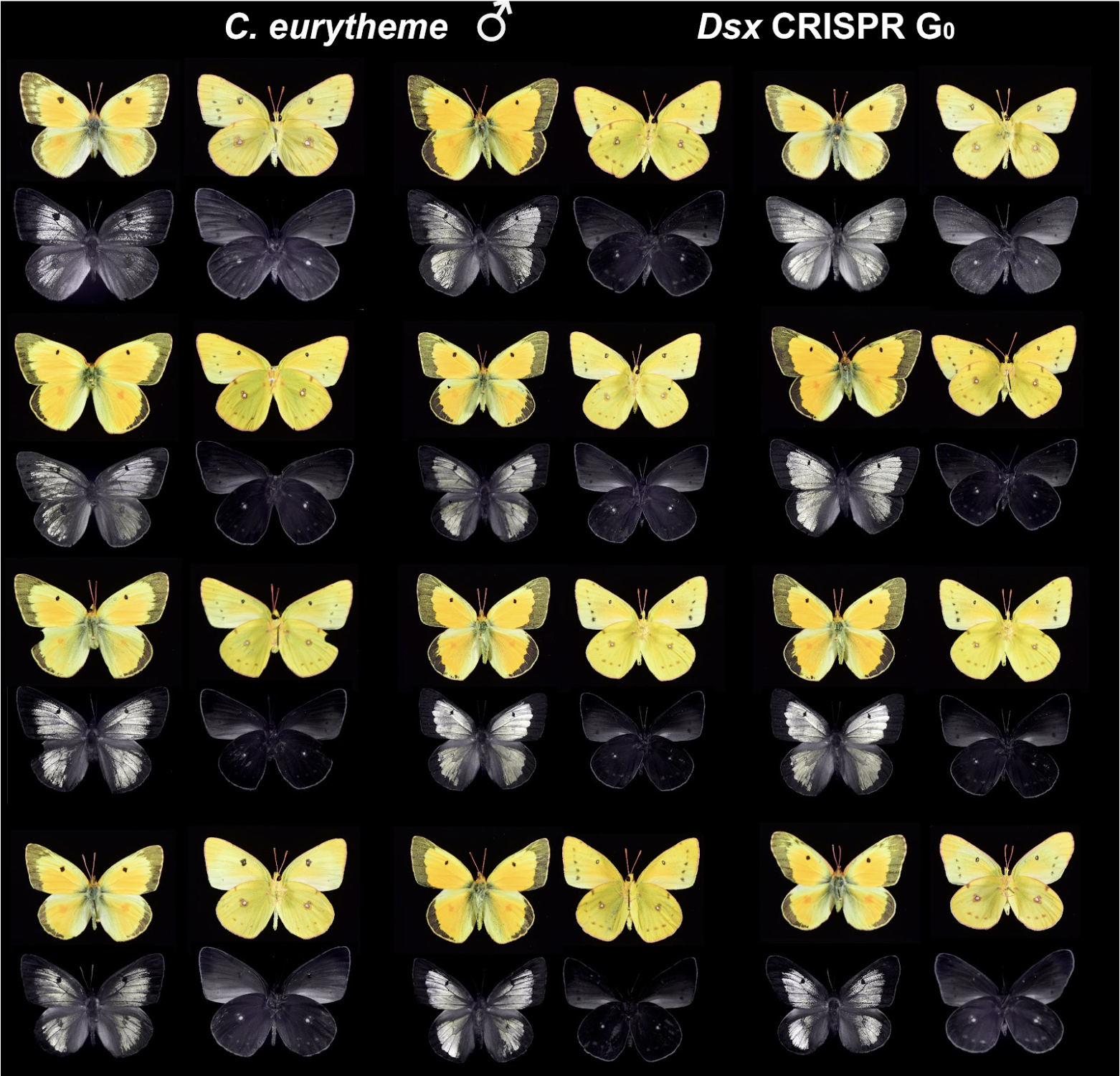

Supplement: S2 Fig — Dsx mosaic knock-out effects are exclusively visible in the marginal section of the male dorsal wings (left panels), with a proximal extension of the melanic band, a proximal regression of the distal border of the UV iridescent region (also visible in the visible spectrum as a yellow extension), and a transformation of scent-related marginal scales into regular melanic scales (not visible here). There were no visible effects on ventral sides (right panels). Bottom rows: UV-spectrum photography (320–400 nm). (JPG) [file pbio.3003233.s002.jpg]

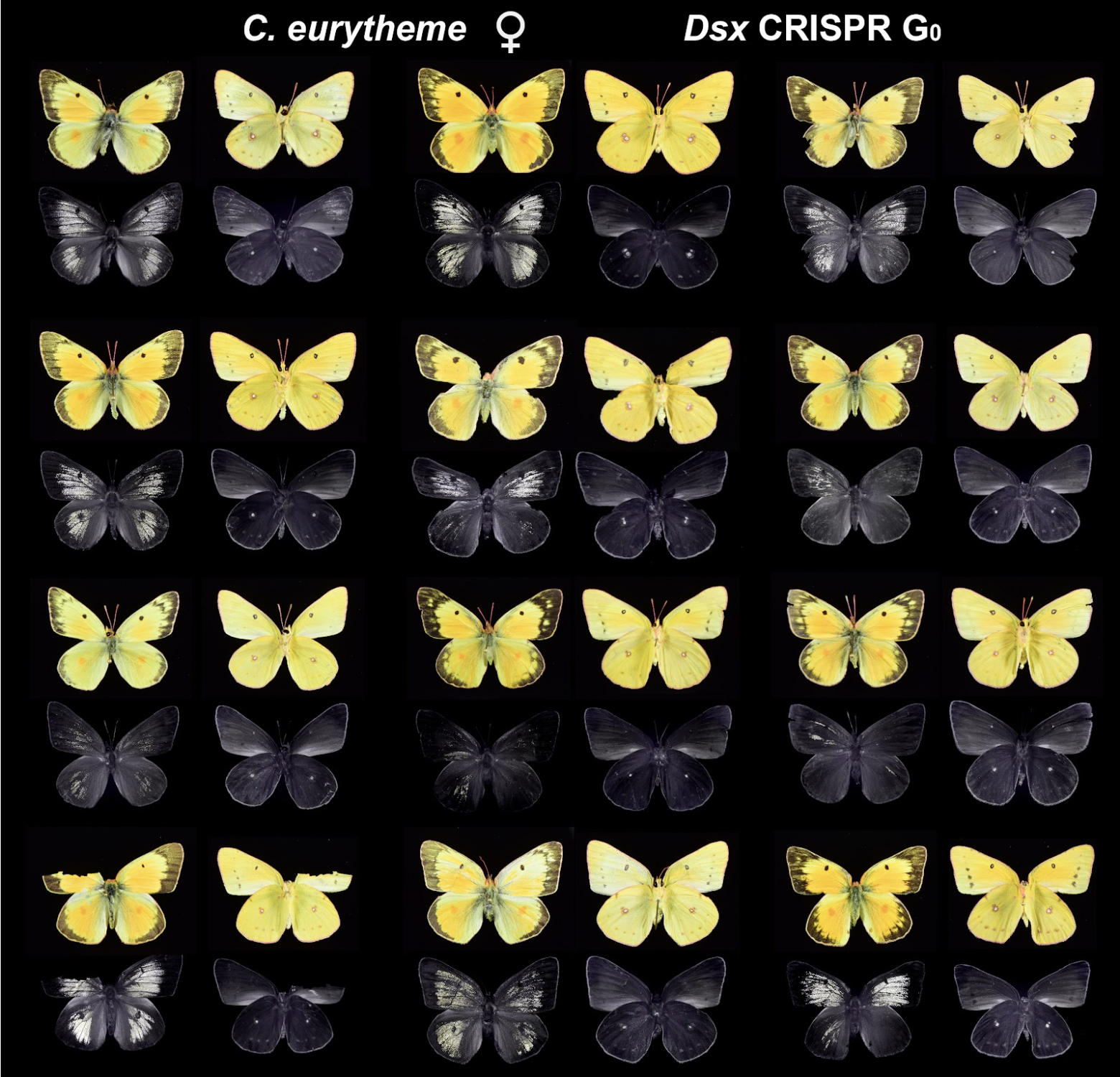

Supplement: S3 Fig — Female Dsx crispants show a male-like regression of the marginal melanic band, a transformation of marginal melanic scales into male-like scent-related scales (not visible here), and widespread gains of UV-iridescence, all restricted to the dorsal side of each specimen (left panels). There were no visible effects on ventral sides (right panels). Bottom rows: UV-spectrum photography (320–400 nm). (JPG) [file pbio.3003233.s003.jpg]

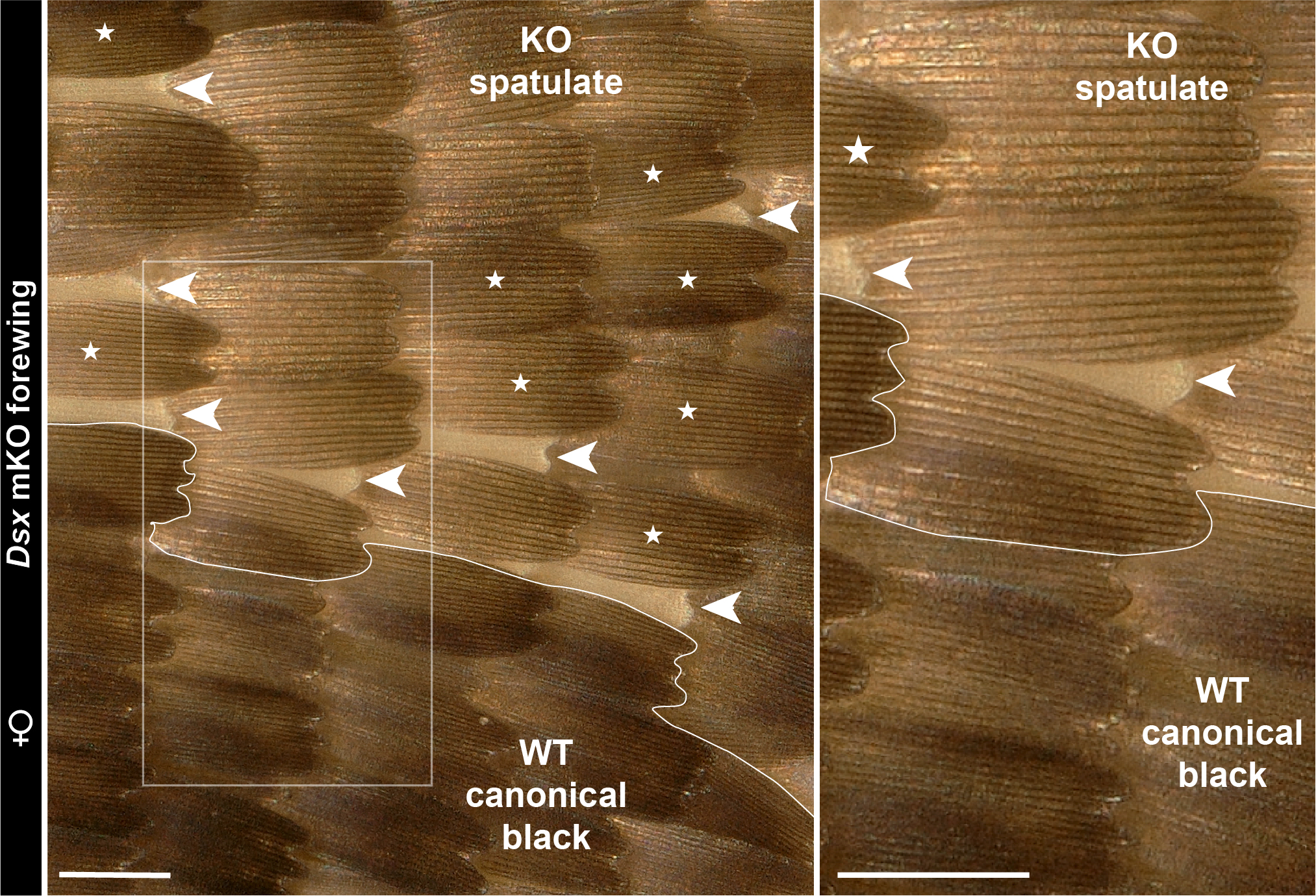

Supplement: S4 Fig — Additional example of a DsxF mKO phenotype in a female dorsal forewing marginal region. The white line delineates the clonal boundary between KO (top) and WT areas. Stars demark scales with intermediate canonical-to-spatulate transformations. The dorsal margins of the female forewing show black ground scales, here transformed to a male-like yellow state in mKO areas (arrowheads). Scale bars: 50 μm. (JPG) [file pbio.3003233.s004.jpg]

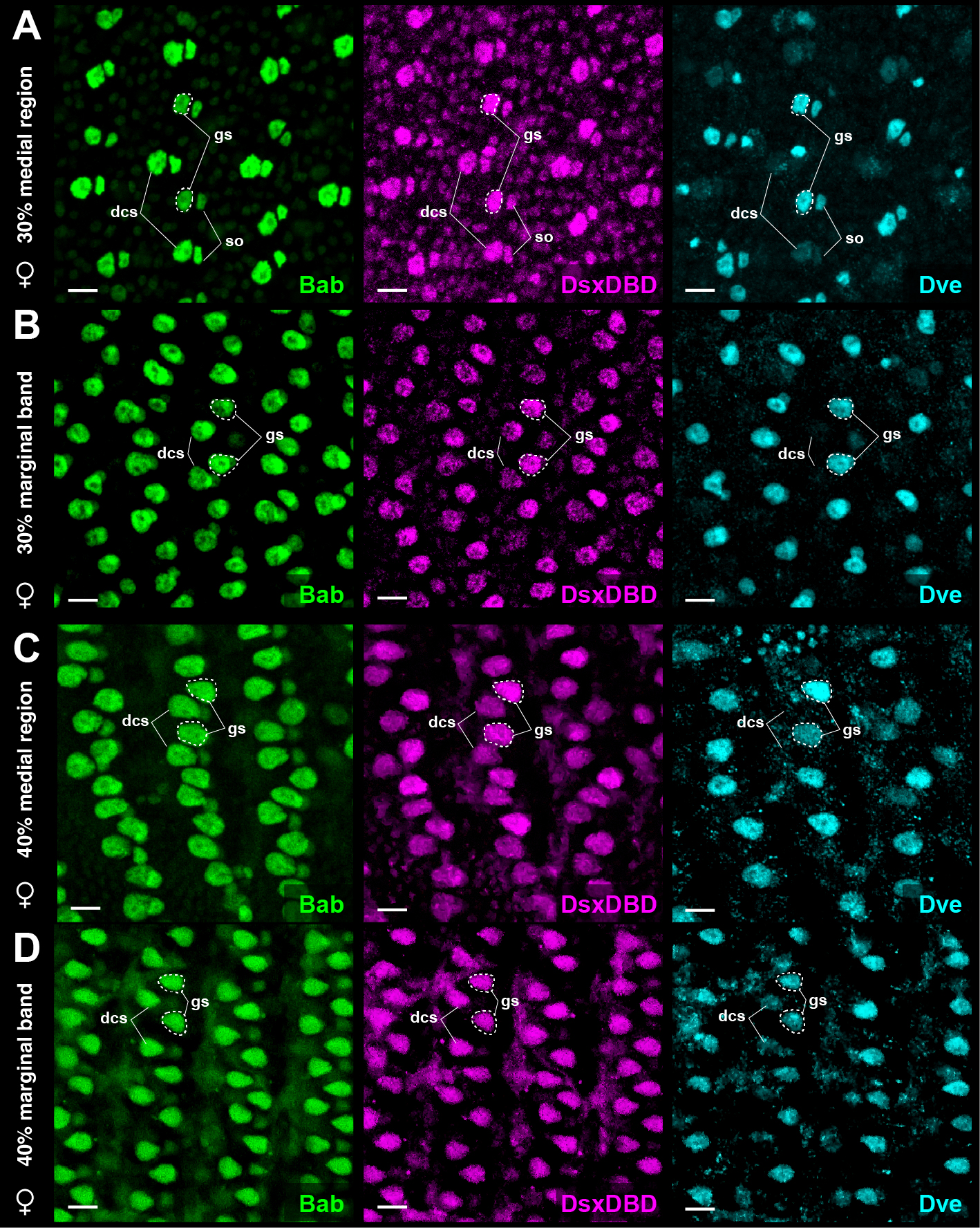

Supplement: S5 Fig — Immunofluorescent detection of the Bab (green) and DsxDBD (magenta) antigens in both the cover scales (dcs) and ground scales (gs) of female wings sampled at the 30% (A, B) and 40% stages (C, D). The Dve antigen (cyan) marks ground scales. Socket cells (so) are visible at the 30% stage in the medial region only (A). Scale bars: 10 μm. (JPG) [file pbio.3003233.s005.jpg]

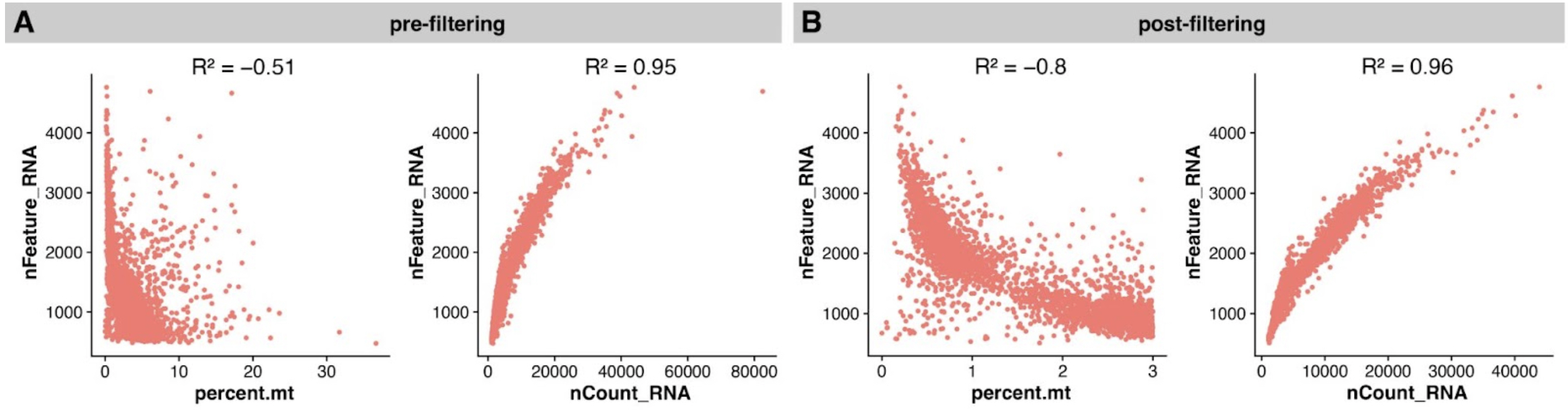

Supplement: S6 Fig — These plots compare CellRanger outputs before (A) and after (B) filtering for nCounts > 3, nFeatures > 300, percent.mt < 4%. Left panels: number of genes detected in each cell relative to percentage mitochondrial reads within each cell. Right panels: number of genes detected in each cell relative to the total number of molecules detected within a cell. R2 values indicate the coefficient of determination in each comparison. (JPG) [file pbio.3003233.s006.jpg]

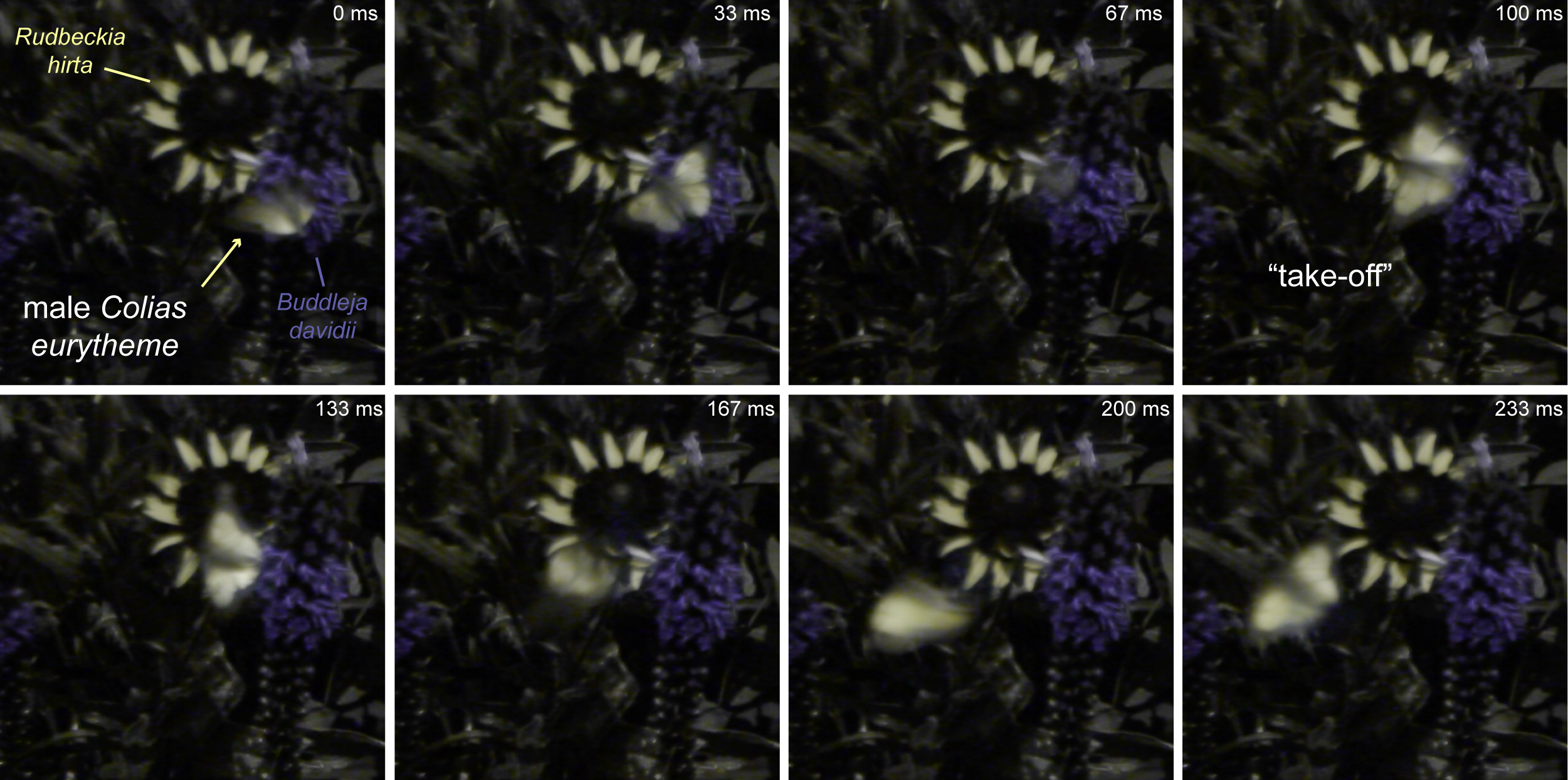

Supplement: S7 Fig — Eight consecutive video frames of a male C. eurytheme butterfly under natural sunlight. UV-A imaging (315–400 nm) was done using a full-spectrum converted Panasonic G3 camera, mounted with a Kyoei-Kuribayashi 35 mm F3.5 lens on a helicoid focusing adapter, and stacked Hoya U-330 and Schott BG39,1.5 mm glass filters eliminating the visible and infrared wavelengths above 400 nm. The butterfly is seen flickering its wings in the first three frames, while resting on a nectaring plant. Take-off and flight are visible on the following frames. Green vegetation is usually UV-absorbing, with the rare exception of pollinator-attracting signals such as the outer rings of Rudbeckia hirta flowers, as shown here. Female C. eurytheme are undetectable with this set-up due to their lack of iridescence. (TIF) [file pbio.3003233.s007.tif]

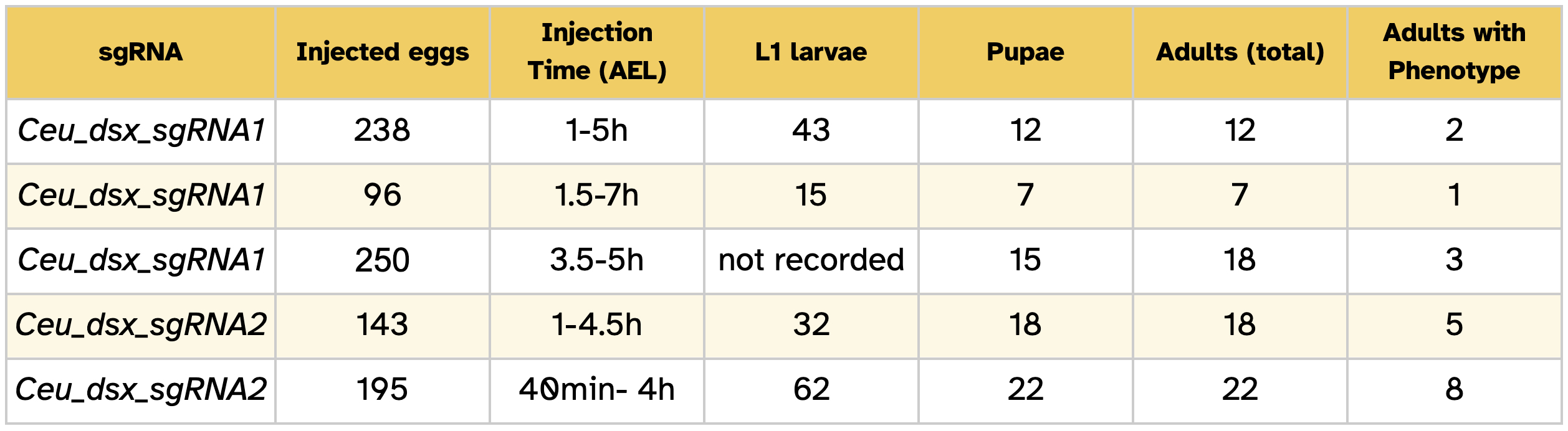

Supplement: S1 Table — (JPG) [file pbio.3003233.s008.jpg]

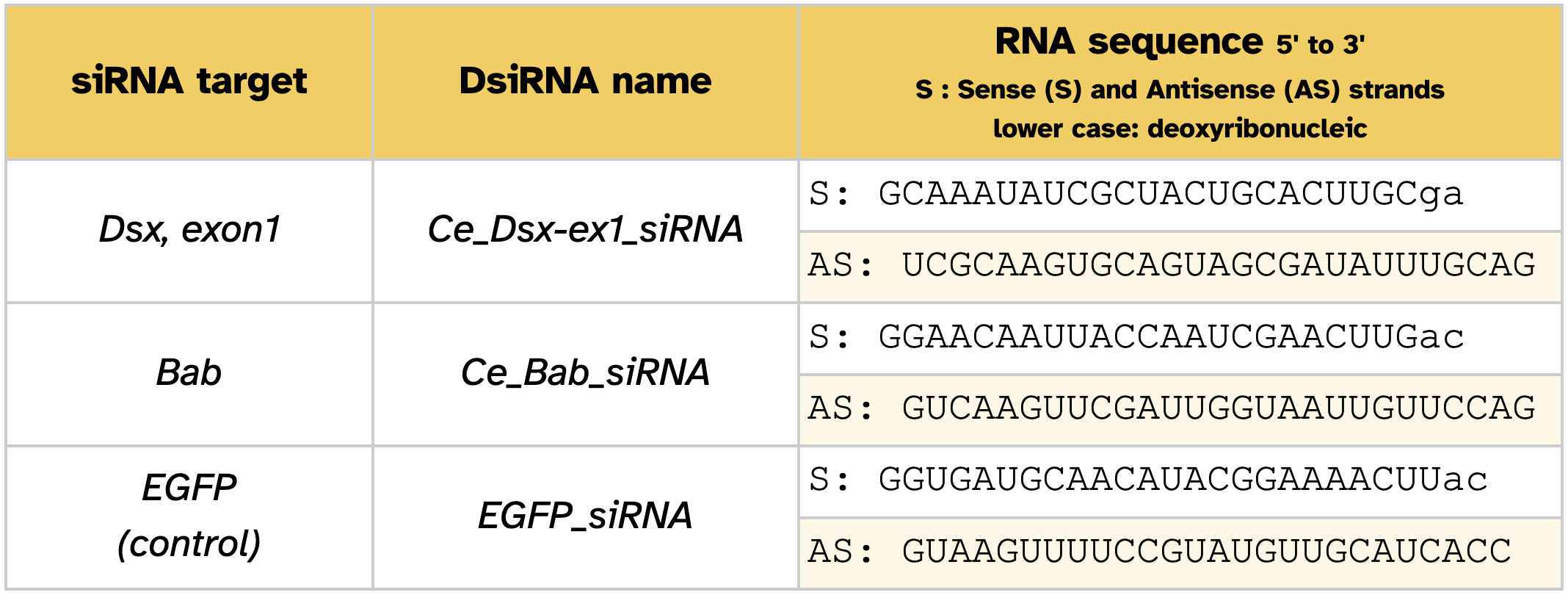

Supplement: S2 Table — (JPG) [file pbio.3003233.s009.jpg]
