# Supplementary material for: Single-nucleus transcriptomics of wing sexual dimorphism and scale cell specialization in sulphur butterflies
Source: PLoS Biol. 2025 Jun 18;23(6):e3003233. doi: 10.1371/journal.pbio.3003233 (PMC12204629; doi:10.1371/journal.pbio.3003233)
Supplement: S1 Text — (PDF) [file pbio.3003233.s010.pdf]

Supplementary Text

<u>Data accessibility and remarks on annotated gene names and marker detection</u>

We provide all necessary data for further exploration of the *Colias* single-nucleus transcriptome in an online repository accompanying this article [1]. This includes the reference genome annotation, inferred gene names, and computed tables for various differential expression analyses. We wish to highlight two technical limitations in our current analysis workflow that can impact future analyses of these datasets.

Gene name annotations do not always indicate direct orthology with traditional model organisms. In our annotation table, we provide two name categories. The first one is based on the best BLASTP hit against the *Drosophila melanogaster* reference protein set. The second one is based on NCBI-provided annotations, derived from the *Colias croceus* annotation. These gene names are based on homology with more phylogenetically distant mammalian cognates. When featuring gene names in our figures, we manually verified reasonable orthology relationships by performing additional BLAST analyses across insects, and favored *Drosophila* gene names when we could identify a one-to-one orthology. Gene names in the provided tables require more caution on a case by case basis, and an insect-wide gene orthology and nomenclature will be required in the future to address these issues.

Lastly, we followed common recommendations to include intronic reads in the definition of transcribed units for our single-nucleus experiment [2–4]. While this allows the detection of transcripts expressed at low levels and is essential for this type of analysis, this means that overlapping gene annotations can create erroneous allocations of a given signal to a gene name in our DE tables. For example, if *Gene A* is differentially expressed but is nested within *Gene B*, *Gene B* may be reported as the DE gene. With this caveat in mind, it will be important to manually examine the genomic context of candidate genes before proceeding to gene-specific assays, and the definitive resolution of this issue will require innovation in RNA mapping pipelines for this type of analysis.

<u>Supplementary methods : PCR genotyping</u>

The chromosomal sex of *Dsx* crispants was determined by genotyping the presence of one or two Z-linked alleles by Sanger sequences. Two Z-linked PCR products (*M13F-fwd1*: 5'-TGTAAAACGACGGCCAGTCTCCGGGATGACTACTTGAC-3'; *rev1* : 5'-TGATCTCCGGAGCCATGAAG-3'; *M13F-fwd2*: 5'-TGTAAAACGACGGCCAGTTTAACACACGACTGAGACCC-3'; *rev2* : 5'-TCGGTGTGAGGCTCAGGTAC-3') were amplified from a single leg using Phire Tissue Direct PCR Master Mix, column-purified, and sequenced using the M13F universal primer. Sequences with double-chromatograms were inferred as originating from ZZ males, while single chromatograms were inferred as females. This method accurately predicted the sex of control individuals of known sex, and matched the inference made from genital morphology in crispant

individuals. Genital morphologies did not show asymmetries or visible phenotypes among surviving adults, a negative result that we attribute to possible lethal effects of *Dsx* CRISPR mKOs during development (**S1 Table**), as previously observed in other butterflies [5,6]. The presence of indel mutations at the CRISPR targets sites was verified using chromatogram deconvolution with ICE [7], on amplicons generated by direct PCR as described above and sequenced with the reverse primer (*Ce_Dsx_sgRNA1*, *fwd* : 5'-GCAATGCTTTTTGCTTGCCA-3', *rev* : 5'-TTTGGTGTTGTCAGGTACGG-3'; *Ce_Dsx_sgRNA2, fwd* : 5'-GTGGGCTTGTGAAATTGCAT-3', *rev* : 5'-GCGTGGGGTCATCCAAAAAT-3').

Supplementary methods : Preparation of Chromatin-Immunoprecipitation (ChIP)-sequencing libraries.

A total of 16 pupal wings were dissected from two females and two males at 40% pupal development for 2 library replicates, and similarly 16 pupal wings from 4 individuals at 60% pupal development for 2 library replicates. Tissues from each individual were fixed in 1% formaldehyde in PBS, before adding PBS with 0.135 M glycine for 5 min, then washing with ice-cold PBS twice. All liquid was removed prior to flash-freezing tissues in liquid nitrogen for 30 s. We performed sonication and library preparation for ChIP-sequencing following previous recommendations [8]. Fixed tissues were dissociated in a sucrose buffer supplemented with Protease Inhibitor (PI) using a dounce homogenizer. Homogenized tissues were then spun at 2,000 *g* for 5 min, and the cell pellet was treated with 1 mL freshly prepared ATAC lysis buffer and PI for 5 min. During this treatment, the cell suspension was pipetted to avoid clumping. Cells in the lysis buffer were spun at 2,000 *g* for five minutes. The lysis buffer was removed, and the pellet was resuspended with 1,000 mL of 1x ChIP Dilution Buffer (Cell Signaling). 150-200 µL of the cell suspension was then distributed into Diagenode 1.5 mL TPX microTubes for sonication. Cell suspensions were then sonicated in < 4°C in a Diagenode Bioruptor UCD-200 (3 x 5 min, 30 s on with *high* setting, 30 s off). Sonicated cell suspensions were then centrifuged at 12,000 *g* to release chromatin, then supernatants from individual tubes were pooled for 20-50 µg of DNA per 1 mL. Sheared DNA was then incubated overnight with 7 µL of 0.550 mg/mL anti-Bab antibody at 4°C, or left untreated for input control samples. Both control and Bab-bound sheared DNA were then treated with 10 µL of Dynabeads Protein A and 10 µL of Protein G (ThermoFisher Scientific Inc. 10001D, 10003D) for a minimum of 2 hours nutating at 4°C. A magnetic rack was used to separate the immunoprecipitated DNA on Dynabeads, washing three times with 1,000 µL of 1x ChIP dilution buffer and twice with 1,000 µL of 1x ChIP Dilution Buffer supplemented with 70 µL of 5M NaCl. Immunoprecipitated DNA on magnetic beads were then resuspended in 1x 150 µL of ChIP Elution Buffer (Cell Signaling Technology) and incubated for 1.5 h at 65°C vortexing every 10 min. After 1.5 h, the supernatant containing the immunoprecipitated DNA was treated with 2 µL of 5M NaCl and 10 µL ProteinaseK, then incubated for 2.5 h to de-crosslink. The samples were then purified using the SimpleChIP Chromatin IP kit (Cell Signaling Technology). DNA libraries were made by normalizing the input to the ChIP-ed DNA, using a NEBNext Ultra Prep II DNA library kit without size selection and with 13 cycles of PCR amplification. Prepared libraries were cleaned with 0.9x Ampure beads

and checked for fragment distribution, before sequencing by the Biotechnology Resource Center (BRC) Genomics Facility (RRID:SCR_021727) at the Cornell Institute of Biotechnology on their Illumina NextSeq 500/550 platform. ChIP-seq libraries were sequenced as PE42 and PE37 reads for samples at 40% and 60% pupal development, respectively, and are available on the NCBI SRA (Bioproject PRJNA1148116).

Supplementary methods : Adult wing imaging. Adult wings were imaged in the visible range using a Nikon D5300 camera mounted with a 105mm f/2.8D AF Micro Nikkor lens, and a VHX-5000 microscope mounted with VH-Z00T and VH-Z100T lenses. For UV-photography, full-spectrum converted Panasonic G3 camera was mounted with UV-transmitting lenses [9,10] and used to image wings under the UV-illumination of GE Blacklights 13-Watt T3 Spiral Light Bulbs and 365nm LED torchlights.

Supplementary methods : Scanning Electron Microscopy and morphometric measurements. Scanning electron microscopy of *C. eurytheme* scales was conducted on dorsal forewing samples taken from one orange female, one Alba female, and two males, following previously described methods [11]. The resulting images were used to select 20-25 scales per scale type categories. Pixel intensity profiles were extracted from transversal sections spanning the visible scale width using *FIJI/ImageJ*, and average ridge distances were measured from these profiles using Fourier analysis implemented in the *SEMolina* code package [12], and as described in a previous publication [13]. Due to inherent differences between scale types, the average number of ridge distances measured per scale varied between 26 ridges for canonical melanic and pterinic scales, 12 for spatulate scales, and 47 for UVI scales.

Supplementary References

1. Loh LS, Martin A. Data for : Single-nucleus transcriptomic signatures of wing sexual dimorphism and scale cell specialization in sulphur butterflies. OSF; 2024. doi:10.17605/OSF.IO/YJVKC

2. McLaughlin CN, Brbić M, Xie Q, Li T, Horns F, Kolluru SS, et al. Single-cell transcriptomes of developing and adult olfactory receptor neurons in Drosophila. Bellen HJ, VijayRaghavan K, Barish S, editors. eLife. 2021;10: e63856. doi:10.7554/eLife.63856

3. Lake BB, Codeluppi S, Yung YC, Gao D, Chun J, Kharchenko PV, et al. A comparative strategy for single-nucleus and single-cell transcriptomes confirms accuracy in predicted cell-type expression from nuclear RNA. Sci Rep. 2017;7: 6031. doi:10.1038/s41598-017-04426-w

4. Bakken TE, Hodge RD, Miller JA, Yao Z, Nguyen TN, Aevermann B, et al. Single-nucleus and single-cell transcriptomes compared in matched cortical cell types. PLOS ONE. 2018;13: e0209648. doi:10.1371/journal.pone.0209648

5. Prakash A, Monteiro A. Doublesex mediates the development of sex-specific pheromone

organs in Bicyclus butterflies via multiple mechanisms. Molecular biology and evolution. 2020;37: 1694–1707.

6. Rodriguez-Caro F, Fenner J, Bhardwaj S, Cole J, Benson C, Colombara AM, et al. Novel doublesex duplication associated with sexually dimorphic development of dogface butterfly wings. Molecular Biology and Evolution. 2021 [cited 2 Aug 2021]. doi:10.1093/molbev/msab228

7. Conant D, Hsiau T, Rossi N, Oki J, Maures T, Waite K, et al. Inference of CRISPR Edits from Sanger Trace Data. The CRISPR Journal. 2022;5: 123–130. doi:10.1089/crispr.2021.0113

8. Lewis JJ, Geltman RC, Pollak PC, Rondem KE, Van Belleghem SM, Hubisz MJ, et al. Parallel evolution of ancient, pleiotropic enhancers underlies butterfly wing pattern mimicry. Proc Natl Acad Sci USA. 2019;116: 24174–24183. doi:10.1073/pnas.1907068116

9. Ficarrotta V, Martin A, Counterman BA, Pyron RA. Early origin and diverse phenotypic implementation of iridescent UV patterns for sexual signaling in pierid butterflies. Evolution. 2023;77: 2619–2630. Available: https://academic.oup.com/evolut/article-abstract/77/12/2619/7292044

10. Ficarrotta V, Hanly JJ, Loh LS, Francescutti CM, Ren A, Tunström K, et al. A genetic switch for male UV iridescence in an incipient species pair of sulphur butterflies. Proceedings of the National Academy of Sciences. 2022;119: e2109255118.

11. Ren A, Day CR, Hanly JJ, Counterman BA, Morehouse N, Martin A. Convergent evolution of broadband reflectors underlies metallic coloration in butterflies. Frontiers in Ecology and Evolution. 2020;8: 206.

12. Hanly J. Hanliconius/semolina. 2020. Available: https://github.com/Hanliconius/semolina

13. Day CR, Hanly JJ, Ren A, Martin A. Sub-micrometer insights into the cytoskeletal dynamics and ultrastructural diversity of butterfly wing scales. Developmental Dynamics. 2019.

Data Files (Data 1-9) are available at the Open Science Framework Repository [1]: **https://osf.io/yjvkc/**

Data 1. Top differentially expressed genes within each cluster in *C. eurytheme* pupal wing at 40% development. List of differentially expressed genes (DEGs) marking each cluster using Seurat's function *FindAllMarkers* (*log2FC* > 0.25, *min.pct* = 0.25, $p < 0.05$, *test.use* = "Wilcox"). Related to **Fig 6**.

Data 2. Top differentially expressed genes defining *Scale2+3* from other *Scale* subclusters in *C. eurytheme* pupal wing at 40% development. List of differentially expressed genes (DEGs) differentiating clusters *Scale2* and *Scale3* from the rest of the clusters using Seurat's function *FindMarkers* (*log2FC* > 0.25, *min.pct* = 0.25, $p < 0.05$, *test.use* = "Wilcox"). Related to **Fig 8**.

Data 3. Top differentially expressed genes defining *Scale2* from *Scale3* in *C. eurytheme* pupal wing at 40% development

List of differentially expressed genes (DEGs) differentiating *Scale2* from *Scale3* using *Seurat*'s function *FindMarkers* (*log2FC* > 0.25, *min.pct* = 0.25, P < 0.05, *test.use* = "Wilcox"). Related to **Fig 8**.

Data 4. Genes with MACS3-called Bab-binding peaks from ChIP-sequencing data from *C. eurytheme* pupal wings at 40% and 60% development

List of genes with Bab-binding peaks mapped using *MACS3* (q value < 0.01) from 2 timepoints, 40% and 60% pupal development with 2 replicates each. Gene list was curated after retaining top N peaks from true replicates below the Irreproducible Discovery Rate (IDR) threshold of 0.05. Related to **Fig 9**.

Data 5. Top differentially expressed genes defining *Scale2+3* from other scale subclusters, with Bab-binding peaks in *C. eurytheme* pupal wing at 40% development

List of genes that are differentially expressed between clusters *Scale2+3* from the rest of the clusters using Seurat's function *FindMarkers* (*log2FC* > 1.25, *min.pct* = 0.01, adjusted $p < 0.05$, *test.use* = "Wilcox") and contain Bab-binding peaks at both 40% and 60% development (False Discovery Rate; FDR < 0.05; **Data 4**). Related to **Fig 9**.

Data 6. Top differentially expressed genes defining *Scale3* from other scale subclusters with several Bab-binding peaks. List of 87 genes that are differentially expressed between clusters *Scale3* from the rest of the clusters using Seurat's function *FindMarkers* (*log2FC* > 1.8, *min.pct* = 0.01, adjusted $p < 0.01$, *test.use* = "Wilcox") and that contain 3 or more Bab-binding peaks (False Discovery Rate; FDR < 0.05; **Data 4**). Related to **Fig 10B**.

Data 7. List of all Bab ChIP sites inside of selective sweeps. List of 29 genomic positions that contain a *C. eurytheme* selective sweep and a Bab ChIP binding site. Related to **Figs 10B, 10D.**

Data 8. List of genes in proximity to Bab ChIP sites and selective sweeps. Related to **Fig 10D.**

Data 9. List of gene accession numbers and annotations in the *C. eurytheme* genome. Gene IDs with corresponding RefSeq annotations from *C. croceus* genome assembly (GCF_905220415.1) using *LiftOff* (Shumate et al., 2020) and available Fly BLAST annotations curated by bidirectional reciprocal blast using *blast+/2.9.0+* and *emboss/6.6.0*.
